# Analysis of the expression of D-dimer, CD147 and miR203 and their correlation in gastric cancer

**DOI:** 10.12669/pjms.35.2.718

**Published:** 2019

**Authors:** Xiguo Wu, Xiuzhen Liu

**Affiliations:** 1 *Xiguo Wu, Department of Laboratory, Affiliated Yidu Central Hospital of Weifang Medical College, Qingzhou, 262500, China*; 2 *Xiuzhen Liu, Department of Functional Inspection, Affiliated Yidu Central Hospital of Weifang Medical College, Qingzhou, 262500, China*

**Keywords:** CD147, D-dimer, Gastric cancer, miR203

## Abstract

**Objectives:**

To evaluate the relationship of D-dimer, CD147 and miR-203, and detect the influence of these biomarkers on the pathological characteristics in patients with gastric cancer.

**Methods:**

The patients with gastric cancer treated using radical gastrectomy between May 2013 and October 2017 were reviewed retrospectively. The expression of D-dimer, miR203 and CD147 was measured for all the patients, and the clinical data including age, gender, tumor size, tumor differentiation, invasion depth, lymphatic metastasis, TMN stage, and pathological type were retrieved and analyzed. The study was carried out in affiliated Yidu Central Hospital of Weifang Medical College, Qingzhou, China.

**Results:**

Two hundred sixty patients with gastric cancer were included. The patients with tumor metastasis, larger tumor diameter, lower differentiation, lymphatic metastasis, deeper invasion, and higher TMN stage presented with a significantly higher D-dimer and CD 147 expression, but the level of the two biomarkers didn’t show a significant difference in patients with different pathological type, gender and age. Compared with CD147 and D-dimer, miR203 presented with different characteristics of expression. In addition, the expression of miR203 was negatively correlated with CD147 and D-dimer, and there was a positive correlation between CD147 and D-dimer in patients with gastric cancer.

**Conclusion:**

In this study, a close correlation of D-dimer, miR203 and CD147 was found, and these three biomarkers should be screened in gastric cancer patients.

## INTRODUCTION

Gastric cancer is the second leading cause of cancer-related death in the world, and its symptoms are mostly non-specific, which accounts for its delay in its early stage.^1^ Subsequently, it is characterized by a poor survival rate due to late diagnosis^2^, and its 5-year survival rate is under 20% for terminal gastric cancer.^3^ As a result, early diagnosis is critical to improve the prognosis of gastric cancer. An exploration of the biomarkers involved in the progression of gastric cancer may facilitate its early diagnosis, and improve its prognosis.

D-dimer is one of the terminal fibrin decomposition products, which can affect cellular signaling systems, promote cell proliferation, stimulate cellular adhesion of tumor cells to endothelial cells and affect platelets and extra-cellular matrix, inducing the growth and spread of tumors.^4^ In a study of 41 gastric cancer patients, Dian analyzed the plasma D-dimer levels and circulating tumor cells, concluded that D-dimer was an essential accompaniment of circulating tumor cells in gastric cancer, which can be used in the detection of hematogenous metastasis.^5^ In another study of 190 patients with different types of cancer, Yu also found gastric cancer patients presented with a high level of plasm D-dimer.^6^ These studies demonstrated that D-dimer was closely correlated with the progression of gastric cancer.

In addition, CD147 and miR203 were also studied by scholars as their close relationship with gastric cancer. CD147, which was isolated from lung carcinoma cells, can increase cancer invasion by inducing MMP synthesis in neighboring fibroblasts, endothelial and cancer cells.^7^ Chu investigated the level of CD147 in 223 gastric cancer patients, and confirmed the association of CD147 with gastric cancer invasion and metastasis, suggesting that CD147 may be an indicator of tumor recurrence and prognosis.^8^ Hu’s study concluded the same conclusion.^9^ In terms of miR-203, its influence on gastric cancer was also studied in recent years, many scholars advocated that the expression of miR-203 was significantly lower in gastric cancer samples than non-cancerous samples, also demonstrating a close correlation between miR-203 and gastric cancer.^10^,^11^

However, few studies have been performed to evaluate the relationship of these biomarkers as well as their influence on the pathological characteristics in patients with gastric cancer. Therefore, in this study we analyzed retrospectively the gastric cancer patients treated surgically in our hospital between May 2013 and October 2017, the aim of our study was:

1. To evaluate the relationship of D-dimer, CD147 and miR-203
2. To detect the influence of these biomarkers on the pathological characteristics in patients with gastric cancer.

## METHODS

In this study, the patients with gastric cancer treated using radical gastrectomy in affiliated Yidu central hospital of Weifang medical college, Qingzhou, China, between May 2013 and October 2017 were reviewed retrospectively.

### The inclusion criteria of this study were

1. The patients diagnosed as gastric cancer by pathological examinations.
2. The patients underwent radical gastrectomy.
3. The patients with pathologic specimens and integrated clinical data.
4. The patients didn’t undergo chemotherapy or radiotherapy before surgery.

### The exclusion criteria were

1. The patients who were pregnant or lactating.
2. The patients with previous malignant diagnoses or concurrent other types of cancer.
3. The patients with a history of thromboembolism, familial coagulopathy or active infections.
4. The patients who received either anticoagulant or anti-aggregate therapies.^12^

The clinical data including age, gender, tumor size, tumor differentiation, invasion depth, lymphatic metastasis, TMN stage, and pathological type were retrieved and analyzed. This study was approved by the Ethics Committee of our hospital.

The expression of D-dimer, miR203 and CD147 was measured for all the patients. Venous blood samples were collected at the second day after admission, and the level of D-dimer was tested using an enzyme-linked fluorescent immunoassay method. The relative expression levels of miR203 were determined using Real-time quantitative PCR. Total RNA was isolated from gastric cancer tissues using the Trizol reagent, cDNA was synthesized from total RNA, and the expression of miR203was tested using a TaqMan miRNA assay.^13^

The level of CD147 was measured by immunohistochemical examination of pathologic specimens. Immunohistochemical staining was performed on 5μm slides from the paraffin-embedded fragments of the gastric cancer specimen. The extent and intensity of immuno positivity were examined and scored. As to the extent, if number of positive stained cell ≤5%, scored 0; 6%∼25%, scored 1; 26%∼50%, scored 2; and >50, scored 3; >75%, scored 4. In terms of the intensity, it was graded as “0” (negative), “1” (weak), “2” (moderate) and “3” (strong). The scores from extent and intensity were multiplied, and the final immunoreactivity scores were defined as high expression if the value>4, or low expression if the value ≤4.^14^

The statistical analysis was conducted using SPSS 21.0 (SPSS Inc., Chicago, IL, United States). Measurement data were presented as mean ± standard deviation and compared using the Student’s t-test. The categorical variables were compared using Chi-squared test, and correlational analyses were performed using Spearman rank correlation analysis. A p value less than 0.05 was regarded as statistical significance.

## RESULTS

In this study, two hundred sixty patients with gastric cancer were included, including 145 males and 115 females. Age ranged from 29 to 86 years old. 95 cases were high or moderate and 165 were low differentiation cancer. 203 cases were adenocarcinoma, 36 were Enjon cell carcinoma, and 21 were other type tumor. In terms of invasion depth, the number of cases in T1, T2, T3 and T4 was 37, 43, 92, and 88, respectively. Lymphatic metastasis was found in 185 cases, and no lymphatic metastasis in 75 cases. Based on TMN stage, 128 cases were I or II stage, 132 were III or IV stage. The clinical characteristics of the included patients showed in Table-I.

**Table-I:** The expression of D-dimer, CD147, MiR203 and related pathological characteristics in 260 gastric cancer patients.

Variable	n	D-dimmer(ug/L)	p	miR203	p	CD147		p

						low	high	
Gender			0.082		0.93			0.532
Male	145	612.9±224.7		0.41±0.18		21	124	
Female	115	545.1±170.9		0.39±0.21		31	84	
Age (year)			0.547		0.59			0.285
<60	108	598.3±176.5		0.38±0.19		25	83	
≥60	152	570.4±169.1		0.42±0.23		27	125	
Tumor metastasis			0.021		0.001			0.000
Yes	198	816.2±432.4		0.31± 0.14		2	196	
No	62	529.3±332.1		0.46±0.24		50	12	
Tumor diameter			0.02		0.001			0.003
>5cm	142	678.5±238.3		0.33±0.09		19	123	
≤5cm	118	312.4±117.6		0.44± 0.15		33	85	
Differentiation			0.012		0.000			0.024
High+ Moderate	95	348.4±141.6		0.47±0.16		40	125	
Low	165	768.3±215.4		0.30±0.11		12	83	
Invasion depth			0.002		0.000			0.000
T1	37	224.5±76.3		0.48±0.16		22	15	
T2	43	286.7±89.5		0.41±0.14		18	25	
T3	92	489.7±125.6		0.35±0.12		10	82	
T4	88	891.5±225.6		0.29±0.08		2	86	
Lymphatic metastasis			0.003		0.001			0.000
Yes	185	863.7±321.4		0.33±0.13		20	165	
No	75	383.7±125.6		0.46±0.17		32	43	
Pathological type			0.098		0.13			0.827
Adenocarcinoma	203	486.7±257.8		0.42±0.18		39	164	
Enjon cell carcinoma	36	312.5±219.3		0.43±0.17		8	28	
Other types	21	419.7±197.9		0.39±0.14		5	16	
Tumor Stage			0.001		0.000			0.047
Stage I+II	128	416.3±156.5		0.44±0.16		32	96	
Stage III+IV	132	879.3±219.4		0.35±0.15		20	112	

***Note:*** low = low expression; high = high expression.

In 260 cases, those with tumor metastasis (p=0.021), larger tumor diameter (p=0.02), lower differentiation (p=0.012), lymphatic metastasis (p=0.003), deeper invasion (p=0.002), and higher TMN stage (p=0.001) presented with a significantly higher D-dimer expression, but the level of D-dimer didn’t show a significant difference in patients with different pathological type (p=0.098), gender (p=0.082), and age (p=0.547) .

The distribution characteristics of CD147 were similar as D-dimer, a significantly higher positive expression was found in patients with tumor metastasis (p=0.000), larger tumor diameter (p=0.003), lower differentiation (p=0.024), lymphatic metastasis (p=0.000), TMN stage (p=0.047), and deeper invasion (p=0.000), but no significant differences in patients with different gender (p=0.532), age (p=0.285), and pathological type (p=0.827) .

Compared with CD147 and D-dimer, miR203 presented with different characteristics of expression. Those with tumor metastasis (p=0.001), larger tumor diameter(p=0.001), lower differentiation (p=0.000), lymphatic metastasis(p=0.001), deeper invasion (p=0.000), and higher TMN stage (p=0.000) presented with a significantly lower expression, but similarly as CD147 and D-dimer, it didn’t show a significant difference in patients with different pathological type (p=0.13), gender (p=0.93), and age (p=0.59) .

Spearman rank correlation analysis demonstrated that the expression of miR203 was negatively correlated with CD147 and D-dimer in patients with gastric cancer (p<0.05), with the expression of miR203 increased, the expression of CD147 and D-dimer decreased. Additionally, there was a positive correlation between CD147 and D-dimer in patients with gastric cancer (p<0.05).

## DISCUSSION

In the current study, we detected the expression of D-dimer, CD147 and miR203, and analyzed their correlation in gastric cancer patients. Few studies have been published in this regard in English literatures. This study may facilitate physicians better predict the progression and prognosis of gastric cancer.

Some studies have been reported to evaluate the expression of D-dimer in gastric cancer. In a study of 247 patients, Liu and colleagues^12^ found that the D-dimer level was correlated with the depth of invasion, lymph node metastasis, peritoneal dissemination, distant metastasis, tumor size and TNM stage by a Spearman correlation analysis. Lin’s study provided a similar conclusion.^15^ In the current study, we also found those patients with tumor metastasis, larger tumor diameter, lower differentiation, lymphatic metastasis, deeper invasion, and higher TMN stage presented with a significantly higher D-dimer expression. Our conclusion confirmed the viewpoints of Liu and Lin. Meanwhile, these studies demonstrated that hypercoagulable state and subsequent hyper fibrinolysis play an important role in the progression and metastasis of malignant tumors.

Moreover, some scholars detected a high expression of CD 147 in gastric cancer. In a meta and bioinformatics analysis, Zheng and colleagues^7^ found that the expression of CD147 was positively correlated with tumor size, depth of invasion, lymph node metastasis, TNM staging and unfavorable prognosis in gastric cancer.^7^ We had the same conclusion in this study. Meanwhile, in Zheng’s study, they found male patients with gastric cancer presented with a higher CD147 expression than the female ones. However, in our study, in 145 male patients, the high expression rate of CD 147 was 85.5%, and in 105 female patients, the rate was 73.0%. Although the rate was higher in male patients, but no significant difference was found between male and female patients. We think if the sample size were enlarged, a significantly different outcome may be found between male and female patients.

Moreover, some scholars have studied the role of miR203 in progression and development of gastric cancer. Chiang^16^ found lower expression of miR-203 tended to have increased tumor sizes and invasion depth in gastric cancer, but no significant difference between the low expression and other clinicopathological characteristics such as sex, age, tumor location, histologic grade and lymph metastasis. In addition, Zhou suggested that the mechanism of miR203 was regulating the level of ataxia telangiectasia mutated kinase-mediated-Snail and E-cadherin^11^, and Hao suggested that it was inducing the overexpression of PIK3CA gene.^17^ Although these two scholars studied the role of miR203 from different angles, they both suggest that miR203 can suppress gastric cancers. In the current study, although we found compared with CD147 and D-dimer, miR203 presented with different expression characteristics in gastric cancer, we didn’t study the mechanism of miR203. Meanwhile, in terms of differentiation and lymphatic metastasis, our conclusion was different from Chiang’s study. The differences may be related to sample size, patient selection or many other factors, which may need further studies to draw a definite conclusion.

At the same time, in this study, we simultaneously investigated the correlation of the expression of CD147, D-dimer and miR203, found the expression of miR203 was negatively correlated with CD147 and D-dimer, and there was a positive correlation between CD147 and D-dimer in patients with gastric cancer. The results demonstrated that the three biomarkers might be used to predict the prognosis and development of gastric cancer significantly. We suggest that the early screen of these biomarkers in gastric cancer patients should be carried out, which may help physicians better understand the severity of gastric cancer, determine the treatment modalities correctly.

### Limitations of the study

First, in the current study, most of our results were consistent with previous studies, but some results were still different. Second, although the close correlation of the three biomarkers was confirmed in the current study, the details of their interactions haven’t been clarified. Third, in the previous published literatures, some studies were performed to explain how these biomarkers affect the progression and prognosis of gastric cancer, but the mechanisms were still unclear. Therefore, more studies need to be carried out to further solve these issues in the future

## CONCLUSION

In conclusion, we evaluated the expression of D-dimer, CD147 and miR203 in this study, and found their close correlation, suggesting the early screen of the three biomarkers should be performed in gastric cancer patients.

### Author`s Contribution

**XGW** conceived, designed and did statistical analysis.

**XGW and XZL** did data collection and manuscript writing.

**XGW** did review and final approval of manuscript.

## References

[1] Zhou B, Wang G, Gao S, Chen Y, Jin C, Wang Z (2017). Expression of ERO1L in gastric cancer and its association with patient prognosis. Exp Ther Med.

[2] Gu Z, Li Y, Yang X, Yu M, Chen Z, Zhao C (2018). Overexpression of CLC-3 is regulated by XRCC5 and is a poor prognostic biomarker for gastric cancer. J Hematol Oncol.

[3] Yu H, Rong L (2018). Emerging role of long non-coding RNA in the development of gastric cancer. World J Gastrointest Oncol.

[4] Liang Y, He D, Wu L, Ding X, Wang X, Wang B (2018). Elevated preoperative plasma D-dimer dose not adversely affect survival of gastric cancer after gastrectomy with curative intent:A propensity score analysis. Chin J Cancer Res.

[5] Diao D, Cheng Y, Song Y, Zhang H, Zhou Z, Dang C (2017). D-dimer is an essential accompaniment of circulating tumor cells in gastric cancer. BMC Cancer.

[6] Yu J, Li D, Lei D, Yuan F, Pei F, Zhang H (2016). Tumor-Specific D-Dimer Concentration Ranges and Influencing Factors:A Cross-Sectional Study. PLoS One.

[7] Zheng HC, Gong BC (2017). CD147 expression was positively linked to aggressiveness and worse prognosis of gastric cancer:a meta and bioinformatics analysis. Oncotarget.

[8] Chu D, Zhu S, Li J, Ji G, Wang W, Wu G (2014). CD147 expression in human gastric cancer is associated with tumor recurrence and prognosis. PLoS One.

[9] Hu C, Dong X, Wu J, Xiao F, Shang J, Liu L (2017). CD147 overexpression may serve as a promising diagnostic and prognostic marker for gastric cancer:evidence from original research and literature. Oncotarget.

[10] Zheng Y, Liu W, Guo L, Yang X (2017). The expression level of miR-203 in patients with gastric cancer and its clinical significance. Pathol Res Pract.

[11] Zhou P, Jiang N, Zhang GX, Sun Q (2016). MiR-203 inhibits tumor invasion and metastasis in gastric cancer by ATM. Acta Biochim Biophys Sin (Shanghai).

[12] Liu L, Zhang X, Yan B, Gu Q, Zhang X, Jiao J (2014). Elevated plasma D-dimer levels correlate with long term survival of gastric cancer patients. PLoS One.

[13] He S, Zhang G, Dong H, Ma M, Sun Q (2016). miR-203 facilitates tumor growth and metastasis by targeting fibroblast growth factor 2 in breast cancer. Onco Targets Ther.

[14] Shen L, Dong X, Yu M, Luo Z, Wu S (2017). beta3GnT8 Promotes Gastric Cancer Invasion by Regulating the Glycosylation of CD147. J Cancer.

[15] Lin X, Wu W, Huang Z, Jin R, Wu F, Chen X (2018). Correlation and its analysis of D-dimer and vWF levels with pathological features in patients with gastric cancer. Chin J Health Lab Tec.

[16] Chiang Y, Song Y, Wang Z, Chen Y, Yue Z, Xu H (2011). Aberrant expression of miR-203 and its clinical significance in gastric and colorectal cancers. J Gastrointest Surg.

[17] Hao Q, Lu X, Liu N, Xue X, Li M, Zhang C (2013). Posttranscriptional deregulation of Src due to aberrant miR34a and miR203 contributes to gastric cancer development. BMB Rep.

